# Challenges and burden of the Coronavirus 2019 (COVID-19) pandemic for child and adolescent mental health: a narrative review to highlight clinical and research needs in the acute phase and the long return to normality

**DOI:** 10.1186/s13034-020-00329-3

**Published:** 2020-05-12

**Authors:** Jörg M. Fegert, Benedetto Vitiello, Paul L. Plener, Vera Clemens

**Affiliations:** 1 Department for Child and Adolescent Psychiatry/Psychotherapy, University of Ulm, Steinhövelstr. 5, 89073 Ulm, Germany; 2 Division of Child Neurology and Psychiatry, Regina Margherita Pediatric Hospital, Department of Public Health and Pediatric Sciences, University of Turin, Turin, Italy; 3 Department for Child and Adolescent Psychiatry, Medical University of Vienna, Vienna, Austria

**Keywords:** Coronavirus disease 2019 (COVID-19), Pandemic, Children, Adolescents, Mental health, Recession, Economic hardship, Adverse childhood experiences, Domestic violence family, SARS-CoV-2

## Abstract

**Background:**

The coronavirus disease 2019 (COVID-19) is profoundly affecting life around the globe. Isolation, contact restrictions and economic shutdown impose a complete change to the psychosocial environment in affected countries. These measures have the potential to threaten the mental health of children and adolescents significantly. Even though the current crisis can bring with it opportunities for personal growth and family cohesion, disadvantages may outweigh these benefits. Anxiety, lack of peer contact and reduced opportunities for stress regulation are main concerns. Another main threat is an increased risk for parental mental illness, domestic violence and child maltreatment. Especially for children and adolescents with special needs or disadvantages, such as disabilities, trauma experiences, already existing mental health problems, migrant background and low socioeconomic status, this may be a particularly challenging time. To maintain regular and emergency child and adolescent psychiatric treatment during the pandemic is a major challenge but is necessary for limiting long-term consequences for the mental health of children and adolescents. Urgent research questions comprise understanding the mental health effects of social distancing and economic pressure, identifying risk and resilience factors, and preventing long-term consequences, including—but not restricted to—child maltreatment. The efficacy of telepsychiatry is another highly relevant issue is to evaluate the efficacy of telehealth and perfect its applications to child and adolescent psychiatry.

**Conclusion:**

There are numerous mental health threats associated with the current pandemic and subsequent restrictions. Child and adolescent psychiatrists must ensure continuity of care during all phases of the pandemic. COVID-19-associated mental health risks will disproportionately hit children and adolescents who are already disadvantaged and marginalized. Research is needed to assess the implications of policies enacted to contain the pandemic on mental health of children and adolescents, and to estimate the risk/benefit ratio of measures such as home schooling, in order to be better prepared for future developments.

## Background

Coronavirus disease 2019 (COVID-19) is profoundly affecting lives around the globe. Isolation, contact restrictions and economic shutdown impose a complete change to the psychosocial environment of affected countries. The current situation affects children, adolescents and their families in an exceptional way. Kindergartens and schools have been closed, social contacts strongly limited and out-of-home leisure time activities canceled. Parents are asked to support their children with home schooling, while at the same time working from home. External support by other family members and social support systems have fallen away. Beside worries and anxieties related to COVID-19, the economic situation has worsened with high and rising levels of unemployment in all affected countries. This has put a lot of pressure on children, adolescents and their families which could result in distress, mental health problems and violence.

As the pandemic is evolving through phases, this paper evaluates the impact these phases might have on mental health of children and adolescents and the provision of psychiatric services. This paper highlights some key challenges and concerns for treatment and research on child and adolescent psychiatry (CAP) across Europe during the different pandemic phases and offers some recommendations that can be adopted immediately.

## Methods

Due to the complexity of the issues and research questions and the aim to provide timely support for CAP professional, as method, a narrative review was chosen. A selective scientific literature review was conducted based on knowledge about the course of epidemics, current experiences in CAP treatment and personal communication with CAP professionals all over Europe [1].

## Main text

### Epidemiological phases of pandemic

**Fig. 1:**
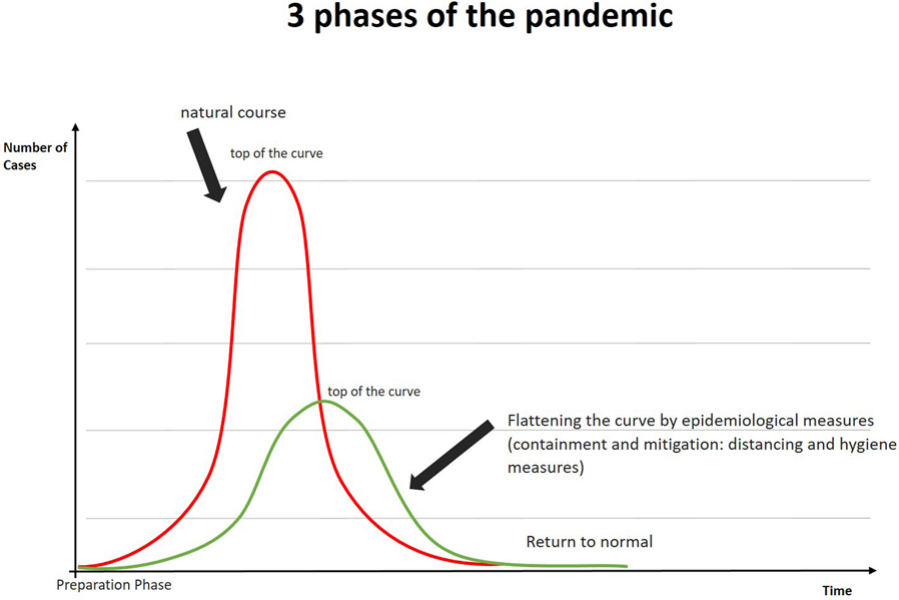
Three phases of the pandemic. Pandemic infections can be divided into three phases: preparation phase, punctum maximum phase and return to normality phase. Within each phase, different psychological reactions exist: Epidemiological measures lead to a flattening and prolongation of the curve

Knowledge of epidemic infections allows us to divide the pandemic into three phases, and to identify, within each phase, different psychological reactions (see Fig. 1).

- Phase one or the preparation phase: Governments enforce social distancing, shutdown and general measures of hygiene in order to contain and mitigate the spreading of the infection and latten the curve of new cases over time.
- Phase two or the punctum maximum phase: The curve reaches the highest incidence of new cases, and mortality rate peaks, including a plateau phase. In the current pandemic, a predictions of when this occurs have been made, and some countries seem to have passed this point while many others have not yet reach it.
- Phase three or the return to normality phase: recovering from the pandemic, which requires re-organizing and re-establishing services and practices.

Measures of containment and mitigation, such as social distancing and hygiene, can succeed in flattening the curve and in this way reducing the height of the punctum maximum (number of infected subjects), but with a more prolonged time course for returning to normality.

## Pandemic-related mental health risks of children and adolescents

### During the pandemic (phase one and two)

During the recent Coronavirus disease 2019 (COVID-19) outbreak in China, 54% of the participants of a large online study rated the impact of the outbreak on their mental health as moderate to severe, with depressive symptoms and anxiety being the conditions most often stated [2]. The current crises imposes multifaceted burdens on children. They include the socio-ecological impact of the pandemic, which is understood to be enormous. The environment of children is affected at different levels– including community and family - as well as the individual child itself [3].

#### Community-related risks for mental health

Since the pandemic was announced, at the community level, there has been disruption of, or more limited access to basic services, such as kindergarten, schools, and routine medical care [4]. Several countries have seen a re-organization of hospital services, with provisional care (including re-assigning doctors and nurses not usually involved in critical care). There have been closures, partial closures or reduced services of inpatient and day-care facilities, with outpatient contacts reduced in some places to emergency cases only. Some hospitals have been unable to accept new inpatients due to the risk of infection [1]. Questions have arisen on how to deal with the risk of infected patients in closed units infecting staff and other patients. There have been concerns for the possible future lack of adequate resources for mental health services as most resources are directed towards ICU and somatic care [1]. Importantly, even the activity of child protection services and currently existing programs of support or supervision by youth welfare agencies have been disrupted or interrupted [5]. The lack of access to these basic services can be particularly harmful for vulnerable children and/or families.

Moreover, leisure time activities have been limited. In most countries, children have not been allowed to use regular playgrounds, social group activities are prohibited and sports clubs are closed [4]. Social relations have been strongly limited to closest family members. In several countries, contact to peers has been prohibited or severely limited [6]. This can have a negative impact on children and adolescents given the importance of peer contact for well-being [7, 8]. Many countries have experienced a lock-down of schools [9]. As pointed out by a recent review, school closures may not have a major impact on reducing infections and preventing deaths [10]. Hence, possible negative consequences such as loss of education time, restricted access to peers and loss of daily structure need to be taken into account when estimating the advantages and disadvantages of this particular measure. Moreover, in some communities, stigmatization of infected children and families may occur.

#### Challenges within the families

At the family level, the pandemic has led to a re-organization of everyday life. All family members have to cope with the stress of quarantine and social distancing. School shutdowns have led to home-schooling and potential postponement of exams. Parents have experienced increased pressure to work from home, to keep jobs and businesses running as well as to take care of schooling children at home at the same time, while caregiver resources including grandparents and the wider family have been restricted. Family connections and support may be disrupted. Fear of losing family members who belong to a risk group can increase. In case of death, the pandemic disrupts the normal bereavement processes of families. Grief and mourning of lost family members, especially in cases where contact with the infected member is restricted or refused, could lead to adjustment problems, post-traumatic stress disorder, depression and even suicide of both, adults and young people [11].

It also has fallen on the parents’ shoulders to inform and explain to children about the COVID-19 pandemic, and to handle fear and anxiety accompanying these uncertain times. All family members may have own fears related to COVID-19. Taken together, this can result in enormous stress and psychological distress for all family members.

The pandemic has major economic implications and puts financial pressure on many families. It has been shown in previous economic recessions that economic pressure, even if not accompanied by social distancing, can pose a severe threat to mental health. Firstly, economic recessions and connected factors such as unemployment, income decline, and unmanageable debts are significantly associated with a decrease of mental well-being, increased rates of several mental disorders, substance-related disorders, and suicidal behaviors [12, 13]—risks that of course also concern parents [14]. The recent recession therefore has added to the fact that low socioeconomic status is a well-known risk factor for poor mental health in children [15]. Mental illness and substance abuse of parents significantly influence parent–child relations [16–18] and increase the risk for mental health problems in children [19].

#### Domestic violence and child maltreatment

Additionally, in economic recessions a significant increase in domestic violence can be seen [20]. Income loss and economic hardship can lead to feelings of economic stress and consequent marital conflict [21, 22]. Quarantine can lead to decreased freedom and privacy, and consequently higher stress. It may also increase existing controlling behaviors by perpetrators as they struggle to regain a sense of control. Exposure to perpetrators is increased, and the possibilities of victims to temporarily escape abusive partners are reduced [23]. In the current COVID-19 crisis, there have been reports from all over the world about a significant increase in domestic violence [24]. UN secretary general António Guterres pointed out a “horrifying global surge in domestic violence” [25]. Exposure to domestic violence again significantly affects mental health of children [26, 27] and has the potential to create long-term consequences [28].

Moreover, a notable increase in physical, emotional and sexualized violence against children during recession has been reported. For example, Huang and colleagues were able to prove a doubling of the incidence of abusive head trauma, a particularly severe form of child abuse associated with a high mortality rate, during the “Great Recession” 2007–2010 [29]. In the literature, an increase of all forms of child maltreatment has been proven during a recession in a wide variety of cultures [30]. Based on these data, for the COVID-19 pandemic, a worldwide increase in the risks for children and adolescents is a plausible assumption. The current reduced societal supervision and lack of access to child protection services is an additional burden.

Taken together, despite a lack of literature specifically addressing the impact of recession on children, existing data point towards threats to mental health of children and adolescents. This is confirmed by one study that directly assessed adolescent mental health during the financial crisis in Greece. The researchers found an increase in mental health problems during the recession [31]. Notably, this study also demonstrates that mental health was impaired disproportionately in the most vulnerable socio-economic groups—adolescents whose families faced more severe economic pressure.

#### Quarantine-associated risks

Besides economic pressure, COVID-19 pandemic-related quarantine in several countries could significantly affect mental health. In a recent review on the psychological impact of quarantine, Samantha Brooks and colleagues pointed out that post-traumatic stress symptoms (PTSS) occur in 28 to 34% and fear in 20% of subjects in quarantine [32]. Additional quarantine-related mental health problems include depression, low mood, irritability, insomnia, anger and emotional exhaustion [32]. Horesh et al. argue that the COVID-19 crisis involves numerous characteristics seen in mass traumatic events so an increase in PTSS during and after the pandemic can be expected [33].

The scarcely available data point towards a detrimental effect of disease-containment measures such as quarantine and isolation on the mental health of children. In a study conducted after the H1N1 and SARS epidemics in Central and North America, criteria for PTSD based on parental reporting were met by 30% of the children who had been isolated or quarantined [34].

Another quarantine-associated threat is an increased risk of online sexual exploitation. Since the beginning of the pandemic, children and adolescents have spent more time online, which may increase the risk of contact with online predators. Due to limited social encounter, children’s outreach to new contacts and groups online has increased. As more adults have been isolated at home, there may also be an extended demand for pornography [35]. Europol has already reported an increase in child pornography since the beginning of the pandemic [36].

The question remains, whether infection with COVID-19 can directly lead to onset or aggravation of mental disorders. Seropositivity to influenza A, B and Coronaviruses has been associated with a history of mood disorders [37]. In addition, onset of psychotic disorders has been reported to be associated with different Coronavirus strains [38].

In summary, phases one and two of the current COVID-19 pandemic represent a dangerous accumulation of risk factors for mental health problems in children and adolescents of enormous proportions: re-organization of family life, massive stress, fear of death of relatives, especially with relation to grandparents and great-grandparents, economic crisis with simultaneous loss of almost all support systems and opportunities for evasion in everyday life, limited access to health services as well as a lack of social stabilization and control from peer groups, teachers at school, and sport activities.

#### Can there be beneficial consequences for mental health from the current crisis?

Together with multiple threats to mental health, the current pandemic could also provide opportunities. When families successfully complete the initial transition phase, the absence of private and business appointments, guests and business trips can bring rest and relaxation into family life. Several external stressors disappear. Mastering the challenges of the COVID-19 crisis together may strengthen the sense of community and cohesion among family members. More time with caregivers can go along with increased social support, which strengthens resilience [39]. In addition, children troubled by school due to bullying or other stressors, can experience the situation of home-schooling as relieving, as a main stressor in their everyday life ceases to exist.

Moreover, mastering current challenges could contribute to personal growth and development. Personal growth is an experience of psychological development as compared with a previous level of functioning or previous attitudes towards life. Thus, successful management of stress and trauma can lead to personal growth, which in turn reinforces the sense of competence and becomes a protective factor for coping with future stressors [40].

However, environmental factors such as socio-demographics, individual social networks and social support affect the outcome of a crisis [41, 42]. Therefore, the opportunity for personal growth may be unequal (see also “Focus on high risk children”). Nevertheless, personal characteristics determine stress-related growth as well. These factors include intrinsic religiousness and positive affectivity. Intrinsic religiousness is suggested to help to find meaning in crisis, the relevance of positive affectivity shows the importance of positive mood and attitude for stress-related growth [42].

### The long way back to normality after the pandemic (phase three)

One major challenge after the pandemic will be to deal with its sequelae. One main consequence will be the economic recession and its implications for mental health of children and their families, as discussed above. During the acute phase of the pandemic, stressors such as social distancing, re-organization of family life, school and businesses, fear of COVID-19 infections, and possibly loss of family members/friends are initially in the forefront. Economic problems may be recognized mainly after the acute phase of the pandemic, although their starting point was in an earlier phase. Some parents might have lost their jobs or businesses, while others might have to deal with an accumulated workload or face major re-organization at work. For children and adolescents, the pressure from school to catch-up for time lost during the acute phase of the pandemic may increase. However, there is evidence that rate and direction of change in macroeconomic conditions rather than actual conditions affect harsh parenting [43]. This suggests that the expectation of a negative economic development is a stronger determinant of negative parental behavior than actual recession, which could point towards the conclusion that harsh parenting and violence will have its climax during the acute phase of the pandemic.

Due to interruption of regular medical services, resources in the health care system may not be enough to overcome previous lack of treatment and supervision. Moreover, not only the accumulation of inadequately treated cases, but furthermore the enhanced need for mental health services might be a problem. The increase of mental health problems in children and their families due to recession and quarantine is discussed above and can be expected to further increase in phase three due to emerging recession. Literature suggests that mental health symptoms will outlast the acute phase of the pandemic. In health-care workers, the risk for alcohol abuse or dependency symptoms was still increased 3 years after quarantine [44]. A quarter of quarantined subjects avoided crowded enclosed places and one-fifth avoided public spaces [45].

Additionally, the increased risk of child maltreatment and household dysfunction may not diminish immediately after the pandemic as several triggers such as economic pressure and mental health problems of parents will last for some time. Moreover, sequelae of pandemic-associated increase of maltreatment of children and adolescents may last for a lifetime. Adverse childhood experiences are known to affect the life of survivors across their life span. Long-term effects include increased risk for numerous mental and physical disorders [46], reduced life quality [47], developmental and cognitive impairments [48, 49], social problems [50] and a reduction of up to 20 years in life expectancy [51].

### Focus on high risk children

The consequences of the pandemic can hit every child. However, there are several indicators that children who are already disadvantaged are at highest risk. First, financial losses will cause increased economic pressure to low-income families due to lack of savings. Second, there may be increased disparities between families with high and low socio-economic status, for example due to differences in parental support for home schooling and leisure activities during the pandemic. Specific modalities such as telemedicine and telepsychiatry may be less accessible for children of low-income families who may not have the resources to use telepsychiatry or to use it in a safe and confidential environment.

Another highly vulnerable group is that of children and adolescents with chronic disorders, for whom important support and therapies may have been reduced or cancelled. For children with intellectual disability, it can be hard to understand the situation and the necessity for the restrictions, with consequent increase in anxiety and agitation. Besides, children with disabilities are at higher risk for child maltreatment [52]. During the pandemic, due to lack of social control and impaired ability to communicate, this risk can increase.

Children and adolescents having experienced adverse events before the pandemic occurred are especially vulnerable for consequences of the COVID-19 crisis. The experience of adverse childhood experiences (ACEs) is associated with higher risk for mental health problems. Maltreatment has been found to be associated with consequent heightened neural response to signals of threat [53]. Moreover, while emotional reactivity is increased, emotion regulation is decreased [54]. This suggests that children and adolescents who have experienced adversity before the pandemic are at higher risk to develop anxiety and adopt dysfunctional strategies to manage the COVID-19-associated challenges.

Another important high-risk group is that of children and adolescents with already existing mental health problems. Most mental disorders require regular psychotherapy and psychiatric treatment. Therefore, lack of access to health services can be particularly detrimental. Severity and outcome of mental disorders could worsen because of delay in prompt diagnosis and treatment. This would be especially problematic for conditions such as early onset schizophrenia, in which early treatment is an important prognostic factor. However, there are more pandemic-associated risks for this group. Taking care of children with mental health problems, in particular externalizing disorders, can be challenging [55]—thus adding to the already increased distress of parents during the pandemic.

With increasing levels of psychopathology, capacity for emotion regulation and adaptive coping is reduced, while maladaptive coping increases [56], a pattern also observed in children and adolescents with a history of child maltreatment [57]. Likewise, children with previously existing psychopathology are at greater risk to show severe worry about political news [58]. Together, this suggests that current COVID-19 crisis-associated stress is particularly harmful for children and adolescents with mental disorders or a history of child maltreatment. On the other hand, stress exposure can enhance already existing psychopathology [59], which can lead to more severe courses of mental disorders—meeting reduced treatment resources. An impact of recession on self-harm has been shown in different studies, especially after the world economic crisis 2008 [60]. As self-harm is predominantly found among adolescents [61], an upsurge of self-injurious and suicidal behavior in youth can be hypothesized as a consequence of the COVID-19 pandemic. This issue should receive urgent attention. Thus, it is likely that the COVID-19 pandemic will lead to an exacerbation of existing mental health disorders as well as contribute to the onset of new stress-related disorders in many, especially children and adolescents with pre-existing vulnerabilities—aggravating pre-existing disadvantages.

Unaccompanied and accompanied refugee minors are a high-risk group for all the aforementioned vulnerabilities—low socioeconomic status, experience of ACEs and mental health problems [62]. Additionally, as some early cases of COVID-19 have been reported in refugee institutions, shelters and camps, the consequent panic and fear of infection can increase the risk of stigmatization of refugees. As many countries are hosting a large number of refugees, and there is a lack of medical and psychiatric specialist care for them, COVID-19-associated mental health risk may disproportionately hit these children and adolescents already disadvantaged and marginalized [63, 64].

## Practical challenges and research questions in Child and Adolescent Psychiatry of associated with the COVID-19 pandemic

### Preparation phase (phase one)

During preparation phase, it is necessary to provide clear and correct information to parents and patients. A good understanding of COVID-19 is important to prevent panic and help comply with the measures for containment and mitigation mandated by the government. Information can include tips for parents on how to talk to children and adolescents about COVID-19, the associated risks and the changes in daily life. Moreover, information on how to manage daily life at home during quarantine and social distancing as well as home schooling can be helpful. Support regarding how to address anxiety and stress in children is important (For information on these issues (see e.g. recommendations of ESCAP [65] and AACAP [66]). Information should be made available to parents regarding how to ensure regular psychotherapeutic and psychiatric treatment and crisis intervention during social distancing and other restrictions of phase one and two.

Parents and patients in need of ongoing medication should be advised by their doctor to stock a certain amount in case of a rupture in stock. This is not regarded as hoarding but taking adequate measures to treat chronically ill patients. Furthermore, doctors could issue extra prescriptions in order to ensure that patients have sufficient medication during phase two of the pandemic.

A good option to maintain treatment during the pandemic is telepsychiatry. During the preparation phase, technical prerequisites should be met and already existing tools can be adapted. Concerns about data protection and high technical protection standards should be addressed and user-friendly systems that can be used by all age groups regardless of abilities shall be established. It has been shown in several studies, reviews and meta-analyses, that online-delivered psychotherapeutic care for children and adolescents is feasible and effective [67–70], so that it can be understood as primary source of standard care in times of restricted physical contact. However, not all children and adolescents may have the technical equipment for telepsychiatry. More importantly, not all may have the opportunity for a safe, confidential space at home.

In most countries, national societies of CAP have taken an active role to face the pandemic. They have been putting forward initiatives to support the continuity of care, to guide parents, teachers, children and health professionals on how to handle the mental issues and to advocate national authorities on the bio-psycho-social impact of the crisis on the life of children and adolescents.

### Punctum maximum phase (phase two)

First, CAP needs to provide services for children and adolescents with mental health disorders. A proportion of children with acute and life-threatening psychiatric disorders require CAP inpatient treatments throughout all phases of the pandemic. Children with pre-existing mental needs, e.g. severe forms of autism, have been deprived of professional support systems meaning families and parents have been left to cope on their own. Due to closure of specialized and complex educational settings for children with developmental problems and multiple handicaps in many countries, problems will exacerbate. CAP institutions should keep contact with patients of special need by using online interventions or phone contact, to avoid disrupting current treatment programs and offer support to caregivers.

Severity and outcome of mental disorders could worsen because of delay in prompt diagnosis and treatment. This would be especially problematic for conditions such as early onset schizophrenia, for which the duration of untreated psychosis has been linked with future level of functioning.

Crisis interventions must be accessible all the time. This support will not only be needed by patients with existing mental health disorders, but also those developing anxiety, depression or adjustment problems and post-traumatic stress disorder as a consequence of losing near relatives during the pandemic. Moreover, there are several additional pandemic-related risks for traumatization. As discussed earlier, domestic violence and child abuse might increase due to extended periods of isolation within an abusive or unsafe home, and less intense supervision from child protection services and lacking support from peers or schools. Although words like “anxiety,” “fear,” and “stress” are constantly mentioned in media, specific peri- and posttraumatic implications of this crisis are not acknowledged [33]. To offer specific help is a central task of CAP. Therefore, several appointments should be arranged for patients with new onset of child psychiatric disorders and adequate care provided. Since there is a shortage of CAP professionals throughout Europe, protection of this scare resource is crucial. Therefore, screening of patients via helplines could be useful as a triage measure to identify the most severe cases. Patients who need to be seen in person due to an acute and severe mental health problem should pass a COVID-19 screening before getting into contact with CAP professionals to assure appropriate safety measures including protective gear. The latter also needs to be existent in handling severely agitated or aggressive patients in emergency psychiatric care. As COVID-19 can spread via saliva, protective measures (in addition to masks, gloves and protective clothing) need to include face shields.

Another important issue regarding CAP treatment during the acute phase of the pandemic are restrictions on parental visits for inpatients and their increased isolation from families and social contacts that can lead to an increased risk of depression, anxiety and suicidal behavior. If inpatients have reduced access to family members due to hygiene restrictions, units should maintain contact with the patient’s family as much as possible in order to adhere to the UN Convention on the Rights of the Child. Moreover, digital communication with the patient’s family should be encouraged.

CAP is an understaffed medical discipline, with a shortage of specialists vis-à-vis mental health needs. Some young child psychiatry trainees have been re-assigned to assist in other medical departments for urgent care during the pandemic [1]. This could leave them at risk of delaying or disrupting their training and qualifications. Alternatively, innovative didactic modalities such as online training and supervision, video presentations, web-based programs and courses can provide trainees in child psychiatry with formative activities. Allowing time spent in providing critical care (if re-assigned) to count towards their overall clinical requirements would be an important step to ensure timely completion of training.

### Long return to normality (phase three)

In the third phase of the pandemic (return to normality), it will be important to quickly re-organize and re-establish lost treatment with patients and develop strategies for CAP to deal with the burden and strain of new patients not seen and not referred during the pandemic. During the acute phase of the pandemic, in several countries only emergencies have been addressed. This has not only been restricted to regular mental health services but has also included child protection services and support for children and families. Offering adequate services to families for dealing with the aftermath of the pandemic is central for reducing long-term consequences for mental health.

The economic crisis brought by the pandemic could have long-term negative consequences leading to increased family conflict, abuse, suicidality and substance abuse. Access to mental health services is needed to cope with the increased demand in times of economic recession [13].

Reports are indicating a decline of people using medical services (e.g. emergency services, general practitioners, calls or demands for psychiatric assessment) for fear of being infected by COVID-19. Consequently, after the crisis there might be a sudden surge of activity to a magnitude possibly overwhelming the capacity of CAP services.

During the acute phase of the pandemic victims of domestic violence and child maltreatment may not be noticed due to lack of social control by peers and staff in school, sport clubs etc. and reduced accessibility of support services. In phase three, due to less parental control and more contact to others, some children and adolescents may confide incidents during the pandemic to others. CAP must be prepared to offer specific treatment for these children and adolescents. CAP professionals must be aware of this increased risk and consequently be sensitive for signs of maltreatment. In case of suspicion, specific anamnesis should be taken, and diagnostic clarification made.

### Further research

The current COVID-19 crisis has imposed numerous restrictions on research. Laboratories have been closed, scientific staff has been working from home, recruitment in studies has been paused. However, this time has brought up numerous interesting research questions that should be addressed within the next months. One major question has been the effect of social distancing on children, adolescents and their families. As stated above, isolation and home schooling can affect mental health in multiple ways. However, this has been the first time in history these measures have been implemented widely and comprehensively. Prevalence of psychiatric disorders including anxiety disorders, depression, posttraumatic stress disorder, suicide attempts and NSSI in minors and parents should be monitored. In longitudinal studies, the effect of different political measures on mental health and well-being should be assessed. Risk and resilience factors for children, adolescents and families could be identified with mixed-method approaches. The role of previous traumatization and other environmental and individual factors on stress regulation and coping can give meaningful insights on stress resilience research. During the current pandemic, risk factors accumulate like under a burning glass. For example, disentangling the real-time impact of parental unemployment or family financial hardship and the effect of social distancing and isolation could give important information in order to estimate the costs of current measures. This would also provide information about the risk–benefit ratio of certain measures to inform decision-makers and better prepare for future scenarios. The effects of the current situation on adverse childhood experiences should be assessed. This includes both household dysfunctions such as mental health or substance abuse of parents, and domestic violence and child maltreatment.

Another issue to be addressed is the means in which children and adolescents to stay in touch with peers during the crisis. The last decade has been characterized by an increasing use of online services to get in touch with peers as well as mating. The pandemic may catalyze this transition for good or for bad. It might ease social encounters but might result in unrealistic high demands on partners, tension between the virtual and the real self and increased distance from “real” life. The current crisis has hit several young people at a pivotal moment of their social development. To assess the effect of this virtualization of social relations on further development of children and adolescents is of great interest.

Furthermore, gender issues and the role of fathers and mothers is an interesting subject. The current Corona crisis has shown that “essential employees” are mostly female [71].

With a lot of creativity and motivation to reorganize mental health care, the pandemic has seen a massive increase in the use of telepsychiatry. It is useful to analyze the interventions used during the pandemic such as telepsychiatry and to determine measures of quality control. Patients, CAP professionals and parents should be surveyed on their experiences of online therapy. Outcomes of telepsychiatry in a real-life clinical outpatient setting should be evaluated and compared to regular face-to-face therapy as current knowledge on the efficacy of online interventions is restricted to controlled intervention studies so far.

## Conclusions

There are numerous pandemic-related mental health risks for children and adolescents. During the acute phase, the main burden has been associated with social distancing, increased pressure on families and reduced access to support services. After the pandemic, economic recession and consequences of anxiety, stress and violence exposure may be predominant issues to meet in CAP care.

Despite the difficult circumstances, CAP professionals shall maintain regular and emergency treatment as far as possible in order to reduce negative consequences for children and adolescents. Flexibility and creativity have been needed to ensure treatment during the different phases of the pandemic. All CAP professionals should be aware of the numerous mental health threats associated with the pandemic.

COVID-19-associated mental health risk has disproportionately hit disadvantaged and marginalized children and adolescents. A special focus should be set on these children in order to prevent aggravating pre-existing disadvantages.

The current pandemic accumulates several risk factors for mental health of children and adolescents. Longitudinal real-time studies can help to disentangle these factors and their relevance for stress-regulating coping processes and mental health. Finally, the current pandemic, stressful and disruptive as it is, could provide the opportunity to introduce innovative approaches to delivering mental health services through telepsychiatry thus possibly fostering a more efficient use of available resources. It will remain to be determined through appropriate research which modalities prove indeed effective and safe.

## Data Availability

Data sharing is not applicable to this article as no datasets were generated or analysed during the current study.
